# Generation and characterization of a humanized *ACE2* rat model for the study of SARS-CoV-2 and COVID-19

**DOI:** 10.3389/fmicb.2025.1680273

**Published:** 2025-10-29

**Authors:** Rachel M. Olson, Henda Nabli, Bettina A. Gentry, Daniel J. Davis, Craig L. Franklin, Mary L. Shaw, Elizabeth C. Bryda

**Affiliations:** ^1^Department of Pathobiology and Integrative Biomedical Sciences, College of Veterinary Medicine, University of Missouri, Columbia, MO, United States; ^2^Laboratory for Infectious Disease Research, University of Missouri, Columbia, MO, United States; ^3^Rat Resource and Research Center, University of Missouri, Columbia, MO, United States; ^4^Animal Modeling Core, University of Missouri, Columbia, MO, United States

**Keywords:** ACE2, rat, SARS-CoV-2, COVID-19, animal model, transgenics

## Abstract

Our goal was to generate new rat strains for the study of human pathogenic viruses such as SARS-CoV-2 that use ACE2 for entry into host cells. An over-expression rat model was generated using random transgenesis to integrate copies of human *ACE2* under the control of the ubiquitously expressed CAG promoter into the rat genome. After transgene copy number, mRNA, and protein expression were confirmed, rats were challenged with the SARS-CoV-2 isolate USA-WA1/2020. Wild type rats are not susceptible to high titer challenge, while rats hemizygous for the *ACE2* transgene lost significant body weight and displayed overt clinical signs of infection. This rat model will advance the understanding of COVID-19 and SARS-CoV-2 pathogenesis as well as accelerate the development of vaccines and antiviral therapies and can serve as an animal model for studies of the physiological role of ACE2.

## Introduction

The COVID-19 pandemic spurred intense efforts to develop effective viral detection methods and to identify and assess new strategies for treatment of and protection against infection. This, in turn, created a need to identify appropriate animal models that could be used to study pathogenesis and to evaluate vaccines and antiviral drugs. In humans, the SARS-CoV-2 viral spike protein facilitates entry into cells via interaction with the angiotensin-converting enzyme 2 (ACE2) protein (Yan et al., 2020; Yang et al., 2020; Zhou et al., 2020). However, due to differences in amino acid sequences important for viral binding to ACE2, mice and rats are not susceptible to SARS-CoV or SARS-CoV-2 in the same way (Li et al., 2004; Leist et al., 2020). To overcome this hurdle, either the rodent or the virus must be adapted. Both strategies have been deployed in mouse models, with different pros and cons to each approach. The most well described transgenic mouse model, the B6.Cg-Tg(K18-*ACE2*)2Prlmn/J mouse was originally engineered to understand the pathogenesis of the structurally similar pathogen SARS-CoV. Several additional humanized mouse models have been engineered in response to the SARS-CoV-2 outbreak that have changed the promotor that drives human *ACE2* expression and/or added additional humanized elements involved in SARS-CoV-2 cellular entry or immunity to viral infection (Zhou et al., 2021; Dedoni et al., 2022; Liu et al., 2024).

To date, however, there are no published reports describing rat models expressing the human *ACE2* gene. Compared to the mouse, some aspects of rat metabolism and physiology are more like human, and it is therefore the preferred species for toxicology, pharmacokinetic, and pharmacodynamic studies for pre-clinical work (Szpirer, 2020; Namdari et al., 2021). Improvements in the genetic tools available to manipulate the rat genome have facilitated our ability to make genetic alterations in rats that were previously only possible in mice. To explore the utility of the rat as an alternative animal model for the study of COVID-19, we generated multiple rat strains/stocks that express human *ACE2* and evaluated their susceptibility to infection with SARS-CoV-2 to determine if these humanized rats could serve as new animal models for the study of the virus.

## Materials and methods

### Cells and virus

Vero E6 (CRL-1586; ATCC) were maintained at 37 °C in high glucose Dulbecco’s modified Eagle medium (DMEM; Cat # 11965092; Gibco) supplemented with 10% serum plus II (Cat # 14009C; Sigma-Aldrich) and 1X GlutaMAX (Cat # 35050061; Gibco). The SARS-CoV-2 isolate USA-WA1/2020 was obtained from BEI resources (NR-52281). SARS-CoV-2 was propagated in Vero E6 cells according to BEI recommendations. Supernatant from infected Vero E6 cells was clarified by centrifugation, aliquoted for single-use, and stored at −80 °C. All work with SARS-CoV-2 was performed in a biosafety level 3 laboratory by trained personnel equipped with powered air-purifying respirators.

### Vertebrate animals

#### Ethics statement

All animal procedures comply with the Office of Laboratory Animal Welfare and the National Institutes of Health Guide for Care and Use of Laboratory Animals and were approved by the University of Missouri Animal Care and Use Committee. All rats used in these studies were reared at the University of Missouri in AAALAC International-approved animal housing facilities. All animals were housed in ventilated cages (Thoren Caging, Hazelton, PA) and kept on a 12:12 light cycle. Food and water were available *ad libitum*.

#### Generation and maintenance of ACE2 rats

The human *ACE2* cDNA was purchased from Origene (Catalog #RG208442) and subcloned into pCAG-loxP-STOP-loxP-ZsGreen (Addgene plasmid #51269) using restriction enzymes *Sal*I and *Sac*I. A linearized gel-purified 4,478 base pair (bp) *Asc*I/*Not*I fragment from this genetically modified plasmid was used for microinjections into embryos at a concentration of 4 ng/μL. Embryos were collected from F344/NCrl rats following standard protocols (Rat Embryo Freezing and Thawing Protocol, n.d.) performed at the Rat Resource and Research Center (Columbia, MO, USA). A total of 151 F344/NCrl zygotes were microinjected and transferred to 10-week-old NTac:SD pseudopregnant female recipients. Of the 6 live pups recovered, 1 female (ID# 057CD) was transgene positive. This founder animal was bred to a F344/NCrl male to confirm germline competency and establish the line [RRRC#946: F344-Tg(CAG-*ACE2*)057Bryd]. The strain has been maintained by backcrossing hemizygous transgene-positive animals to F344/NCrl mates and intercrossing hemizygous animals to generate homozygous transgene-positive animals as needed.

A 2,069 bp fragment of the human ACE2 promoter sequence was cloned in place of the CAG promoter in the plasmid used to create line RRRC#946: F344-Tg(CAG-ACE2)057Bryd. This 2,069 bp fragment was cloned via In-Fusion cloning per manufacturer’s instructions using the following In-Fusion cloning primers: 5′ TGGCGCGCCGGATTCTGTGTAGAAATTTATTTC CGCTTTTATTACAGTAACAT 3′ and 5′ GGAAGAGCTTGAC ATCGTCCCCTGTGA 3′. A 4,809 bp linearized gel-purified AscI/NotI fragment from this completed plasmid was used for the microinjections into embryos at a concentration of 4 ng/μL. A total of 226 Crl:CD(SD) zygotes were microinjected and transferred to 10-week-old NTac:SD pseudopregnant female recipients. Of the 53 live pups recovered, there were 4 transgene-positive animals. Two transgene-positive rats, 1 female (ID# 955CP) and 1 male (ID# 058CV) were bred to Crl:CD(SD) mates to confirm germline competency and establish the lines [RRRC#1001: SD-Tg(ACE2)955CPBryd and RRRC#1012: SD-Tg(ACE2)058CVBryd, respectively]. These two stocks have been maintained by outcrossing hemizygous transgene-positive animals to Crl:CD(SD) mates and intercrossing hemizygous animals to generate homozygous transgene-positive animals as needed. All rat strains/stocks are available from the Rat Resource and Research Center (www.rrrc.us).

### Genotype and transgene copy number analysis

Genomic DNA was extracted from tissue samples (e.g., tail snips or ear punches) placed in 180 μL of 50 mM NaOH in a 0.2 mL tube. Samples were incubated at 95 °C for 10 min, 40 °C for 2 min, then cooled to 4 °C. Twenty μL of 1M Tris-HCl (pH 8.0) was added and the sample mixed well by flicking gently.

PCR primers hACE2 JF 5′-CCTGGCTGAAAGACCAGAAC-3′ and hACE2 JR 5′-TCAAATTAGCCACTCGCACA-3′ located upstream of human *ACE2* gene exon 7 and within exon 8, respectively, were used for genotyping F344-Tg(CAG-*ACE2*)057Bryd rats. PCR primers hACE2prom F1 5′-CCCAACCCAAGTTCAAAGGCTG-3′ and hACE2cDNA R1 5′-GAACAGGTCTTCGGCTTCGTG-3′ located in the human *ACE2* promoter sequence and within the human *ACE2* cDNA sequence, respectively, were used for genotyping SD-Tg(*ACE2*)955CPBryd and SD-Tg(*ACE2*)058CVBryd rats. The amplicon sizes were ∼235 bp for the first assay and ∼258 bp for the second assay. The assays were designed to distinguish transgene positive from transgene negative animals but do not distinguish hemizygous from homozygous animals. PCR reactions (20 μL total volume) included 10–20 ng genomic DNA, 10 μL 2X ExtremeTaq HiFi Supermix (Azura Genomics), and 0.3 μL each of 25 μM primers. Reactions were performed in 200 μL thin-walled PCR tubes using a T100 Thermal Cycler (Bio-Rad). Cycling parameters were 1 cycle of 95 °C, 3 min followed by 35 cycles of 94 °C, 30 s; 64 °C, 30 s; 72 °C, 30 s and 1 cycle of 72 °C, 5 min. PCR amplicons were analyzed using the QIAxcel Advanced capillary electrophoresis system (QIAGEN) with the QIAxcel DNA Screening Kit, QX Alignment Marker 15 bp–3 kb, and QX DNA Size Marker 100 bp–2.5 kb. The AM320 injection method with injection of 10 s at 5 kV and separation of 320 s at 6 kV was used.

To determine the number of transgene copies that had integrated in each strain/stock and assess zygosity (hemizygous vs. homozygous), droplet digital PCR (ddPCR) was used. Genomic DNA was extracted from rat tissue using the DNeasy Blood and Tissue Kit (QIAGEN), following the manufacturer’s spin-column protocol for animal tissues The DNA was quantified using a Nanodrop 8000 spectrophotometer (ThermoFisher Scientific) and diluted with nuclease-free H_2_O to a working concentration of 10 ng/μl. Primers to detect the *ACE2* transgene were 5′-GAACCCTGGACCCTAGCATTG-3′ and 5′-GGACTCCAGTCGGTACTCCATC-3′ located in *ACE2* exon 14. The *ACE2* transgene probe was 5′-CCTGGCTGAAAGACCAGAACAAGAATTCT-3′. Primers to detect the rat Rhodopsin (*Rho*) gene which was used as the reference gene were 5′-CTGCGGCAAGAATCCACTG-3′ and 5′-CAGTCTCTGGCCAGGCTTAG-3′ located in Rho exon 5. The Rho probe was 5′-TGCCACTGCCTCCAAGACGGA-3′. The probes were labeled on the 5′ end with 6-FAM and on the 3′ end with Zen-3′Iowa Black FQ. Each 26 μl ddPCR reaction consisted of 13 μl BioRad ddPCR Supermix for Probes (no dUTPs), 0.5 μl of each 25 mM ACE2 transgene primer, 0.5 μl of each 25 mM Rho primer, 0.25 μl of the 20 mM ACE2 transgene probe, 0.25 μl of the 20 mM Rho probe, 0.25 μl of MseI (5 U/μl), 7.25 μl of nuclease-free H_2_O, and 3 μl of 10 ng/μl DNA. Pipetting was performed using Rainin LTS 200 μl filter tips to prevent destruction of droplets. Droplets were generated using the BioRad QX200 Droplet Generator, and reactions were run in a BioRad C1000 Touch Thermal Cycler with 96-Deep Well Reaction Module. Thermal cycler parameters were 1 cycle at 95 °C for 10 min, 40 cycles of 94 °C for 30 s (ramp rate of 2 °C/s) and 60 °C for 1 min (ramp rate of 2 °C/s), and 1 cycle of 98 °C for 10 min. After cycling, the plate was loaded into the BioRad QX200 Droplet Reader, and BioRad QuantaSoft software, version 1.7.4.0917, was used to analyze droplet fluorescence. This data was transferred to the BioRad QuantaSoft Analysis Pro software, version 1.0.596, for data analysis.

### *ACE2* mRNA analysis

Rats were euthanized with CO_2_ overdose followed by bilateral pneumothorax. Brain, kidney, and lung tissues were collected, snap frozen in liquid nitrogen, then stored at −80 °C until further processing. RNA extraction was performed with the RNeasy Mini kit (QIAGEN). Frozen brain tissues (20 mg) were mixed with the supplied buffer RLT with added 1% β-mercaptoethanol (Sigma Aldrich) in 2 ml tubes containing 5 mm stainless steel beads (Penn ball Bearing Company) then homogenized with the TissueLyser II (QIAGEN) at 20 oscillations/s for 2 min. The homogenates were further processed for RNA isolation following the manufacturer’s instructions. An on-column DNase digestion was included to remove genomic DNA using the RNase-Free DNase Set (QIAGEN). Purified RNA was eluted with RNase-free water and the concentration and purity were assessed with a NanoDrop 8000 Spectrophotometer (ThermoFisher Scientific). RNA samples were stored in aliquots at −80 °C until further analysis. ACE2 mRNA in rat tissues was analyzed by RT-qPCR. The cDNA was generated with the Superscript^®^ IV First-Strand Synthesis System kit (Invitrogen) using 1 μg of total RNA and 50 ng/μl random hexamer primers. For gene amplification, PrimeTime™ Predesigned qPCR Assays for ACE2 (Assay Hs.PT.58.27645939; Exon location 14–15; primer1 5′-GCCACTGCTCAACTACTTTG-3′; primer 2 5′-GCTTATCCTCACTTTGATGCT TTG-3′) and Hprt1 reference gene (Assay Rn.PT.39a.22214832; Exon location 7−9; primer1 5′-GGT GAAAAGGACCTCTCGAAG-3′; primer2 5′-GCTTTTCCACTTTCGCTGATG-3′) were obtained from IDT (Integrated DNA Technologies Inc.). Each PCR reaction included 1.0 μl cDNA, 0.5 μl primer mix, 10 μl SsoAdvanced Universal SYBR Green Supermix (Bio-Rad), and nuclease free water (Sigma Aldrich) for a final volume of 20 μl. The PCR was run on the CFX96 Real-Time PCR Detection System (Bio-Rad) with the following parameters: 1 cycle (95 °C for 30 s), 39 cycles of 95 °C at 10 s; 60 °C, 30 s, and a final melt-curve analysis step with the instrument default settings.

### ACE2 protein expression by western blot analysis

Brain, kidney, and lung tissues were homogenized in RIPA buffer consisting of 150 mM sodium chloride, 5 mM EDTA, 50 mM Tris-HCl, 0.5% deoxycholic acid, 0.1% sodium lauryl sulfate and 1% IGEPAL (ThermoFisher Scientific) and fresh protease inhibitor cocktail (Sigma-Aldrich) with the TissueLyser II (QIAGEN) at an oscillation frequency of 20 Hz for 2 min. Supernatants containing total protein were collected following centrifugation of the homogenates at 1,000 *g* for 20 min at 4 °C. Protein concentration was measured with the Pierce™ BCA Protein Assay Kit (ThermoFisher Scientific) following the manufacturer’s instructions. Absorbance was measured at 562 nm on the SpectraMax M3 microplate reader and data was analyzed with the SoftMax Pro 6.2 software (Molecular Devices).

Total protein from rat tissue (100 μg) and human liver carcinoma HepG2 cells (30 μg; Abcam) were mixed with sample buffer (GenScript), heated at 95 °C for 5 min then loaded on an 4%–20% PAGE gel (GenScript) along with molecular size standards (Bio-Rad) and separated by electrophoresis at 140 V for 45 min in Tris-MOPS-SDS running buffer (GenScript). The HepG2 cell lysates served as a positive control. Proteins were transferred to polyvinylidene difluoride (PVDF) membrane (Bio-Rad) at 100 V for 60 min in transfer buffer (GenScript). The membrane was then rinsed with milli-Q water for 5 min and incubated in blocking buffer (20 mM Tris, 150 mM NaCl, 0.05% Tween 20, 5% non-fat milk; pH 7.6) with continuous shaking for 1 h at room temperature then rinsed with wash buffer (20 mM Tris, 150 mM NaCl, 0.05% Tween 20, pH 7.6) for 5 min prior to antibody blotting. The membrane was sectioned transversally in 2 parts with the top section containing the ACE2 protein and the bottom section containing Hprt1 (loading control). The membrane was blotted with anti-ACE2 (1:1,000 dilution in blocking buffer; Abcam) and anti-Hprt1 (1:10,000 dilution in blocking buffer; Abcam), respectively, at 4 °C overnight with continuous shaking. Membranes were washed 5 times in wash buffer for 5 min per wash, incubated in anti-rabbit IgG, HRP-linked antibody (1:1,000 dilution in blocking buffer; Cell Signaling Technology) for 1 h with continuous shaking then rinsed 6 times with wash buffer for 5 min per wash. Protein detection was performed with the SuperSignal^®^ West Atto Chemiluminescent Substrate kit (ThermoFisher Scientific) following the manufacturer’s instructions. Protein bands were visualized on the ChemiDoc™ XRS+ System with Image Lab™ Software #1708265 (Bio-Rad).

### SARS-CoV-2 challenge

For challenge experiments, male and female rats 6–12 weeks of age, were transferred to the Laboratory for Infectious Disease Research (Columbia, MO, USA). All work was conducted at biosafety level 3 by highly trained personnel. Rats were lightly anesthetized with isoflurane and challenged by intranasal instillation of 100 μL of viral stocks diluted in sterile PBS to the concentrations indicated in the figure legends. Rats were then returned to their home cage and monitored until they recovered from anesthesia (generally within 1–2 min of being removed from the induction box). All infected rats were monitored with daily assignment of health scores, which involved assessments of their appearance, activity, and weight. Animals that survived to the end of the study period or were identified as moribund (defined by pronounced neurologic signs, inactivity, severe weakness, or severe weight loss) were euthanized by CO_2_ asphyxiation followed by bilateral pneumothorax or cervical dislocation, methods approved by the American Veterinary Medical Association Guidelines on Euthanasia.

### Histopathology

Tissues from SARS-CoV-2 challenged F344-Tg(CAG-*ACE2*)057Bryd rats were collected at day 6 post challenge (day 10 for select wild type rats). Tissues were placed in 10% neutral buffered formalin for the inactivation of virus for at least 7 days, according to methods approved by the University of Missouri Institutional Biosafety Committee. Sections of lung, brain and decalcified heads (for nasal cavity and eye analysis; low dose group only) were trimmed, embedded in paraffin, and hematoxylin and eosin (H&E)-stained sections were prepared by the histology services laboratory of IDEXX BioAnalytics (Columbia, MO, USA). Histological examination was performed by two blinded board-certified laboratory animal veterinarians experienced in reviewing rodent tissues (B.A.G. and C.L.F.). Slides were randomly ordered so that reviewers were blinded to dose and genotype.

To further characterize encephalitis seen in rats inoculated with the high dose of virus, immunohistochemical (IHC) assays were performed on brain sections by the University of Missouri Veterinary Medical Diagnostic Laboratory (Columbia, MO, USA). The following assays were included: proliferating cell nuclear antigen (PCNA) to assess apoptosis, CD3 to identify T cells, paired box (PAX5) to identify B cells, calbindin to enumerate Purkinje cells, ionized calcium binding adaptor molecule 1 (IBA1) to assess microglial cell activation and glial fibrillary acidic protein (GFAP) to identify astrocytes.

### Statistical analysis

Data from all replicates were analyzed by the statistical test indicated in individual figure legends using GraphPad Prism 10 software (GraphPad Software).

### Diversity statement

We support inclusive, diverse, and equitable conduct of research. We worked to ensure sex balance in the selection of non-human subjects. One or more of the authors of this manuscript self-identifies as a member of the LGBTQ+ community. While citing references scientifically relevant for this work, we also actively worked to promote gender balance in our reference list.

## Results

### Generation and molecular characterization of *ACE2* rats

A random transgenesis strategy was used to generate human ACE2-expressing (*ACE2*) rats on either the Fischer 344 (F344/NCrl) or the Sprague Dawley (Crl:CD) genetic backgrounds. DNA transgene constructs designed to express *ACE2* either under control of the ubiquitously expressing CAG promoter or the human *ACE2* promoter (Table 1) were delivered into rat zygotes by pronuclear microinjection. Manipulated embryos were transferred to surrogate dams, and the resulting offspring were screened for random integration of the transgene using a standard endpoint PCR assay. Three independent transgene positive founders were used to establish the three strains/stocks. F344-Tg(CAG-*ACE2*)057Bryd is on the inbred F344 genetic background and carries the CAG-*ACE2* transgene. SD-Tg(*ACE2*)955CPBryd and SD-Tg(*ACE2*)058CVBryd are on the SD outbred genetic background and carry the transgene with the human *ACE2* promoter driving expression of *ACE2*. While the exact genomic site where the transgene integrated is currently unknown in these three lines, we used a droplet digital PCR assay to determine how many copies of the transgene are present at the genomic integration sites in each strain/stock. Hemizygous F344-Tg(CAG-*ACE2*)057Bryd (RRRC#946) rats carry a single copy of the CAG-*ACE2* transgene. Hemizygous SD-Tg(*ACE2*)955CPBryd (RRRC#1001) rats carry 30 copies and hemizygous SD-Tg(*ACE2*)058CVBryd (RRRC#1012) rats carry 8 copies of the *ACE2* transgene (Table 1).

**TABLE 1:** Genetic characteristics of transgenic *ACE2* rats.

RRRC strain ID^#^	Background strain/stock	Strain/stock name	Promoter	Transgene copy^#^
946	Fischer 344	F344-Tg(CAG-*ACE2*)057Bryd	CAG	1
1001	Sprague Dawley	SD-Tg(*ACE2*)955CPBryd	ACE2	30
1012	Sprague Dawley	SD-Tg(*ACE2*)058CVBryd	ACE2	8

^#^RRRC strain ID.

**FIGURE 1:**
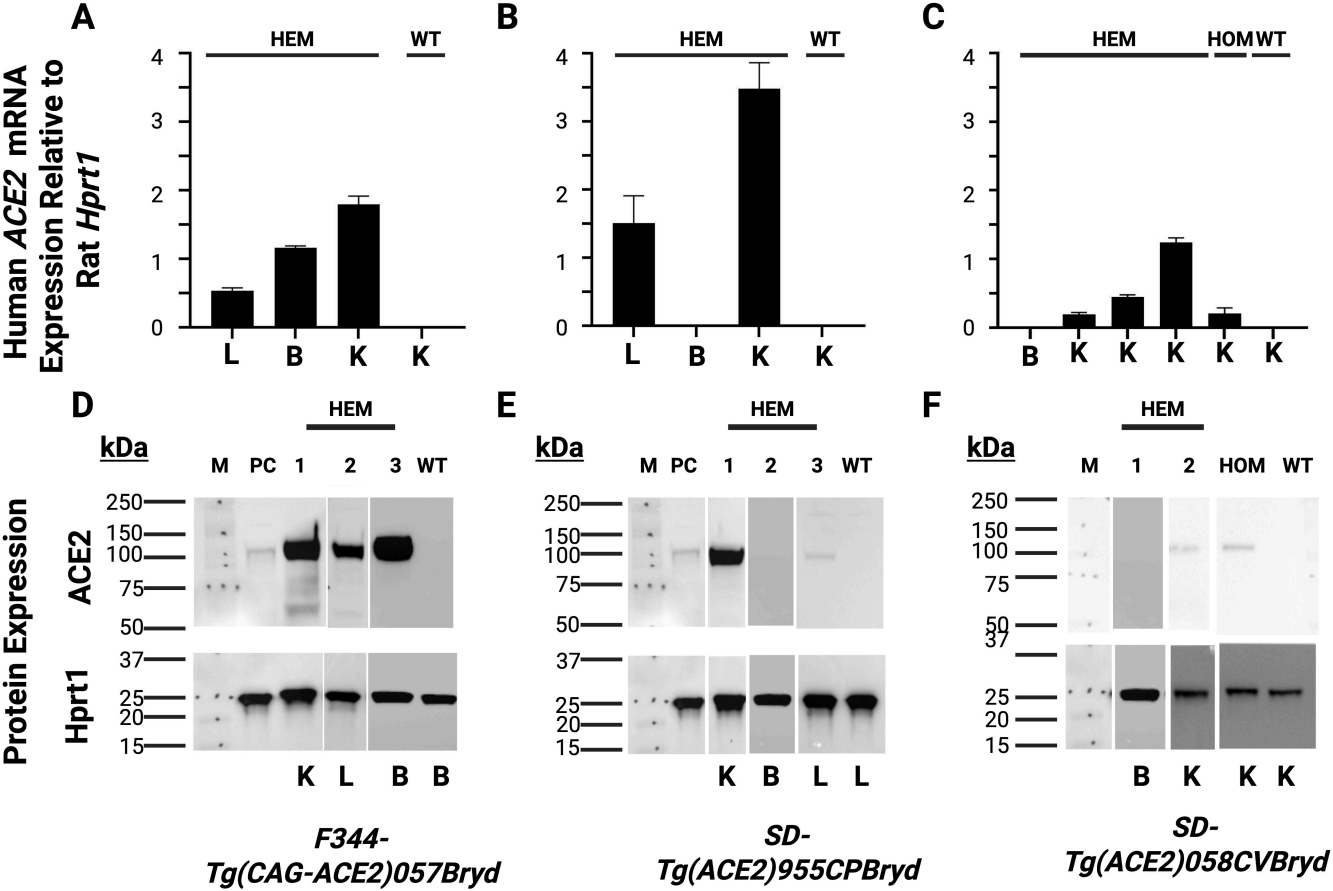
Relative mRNA detection and western blot analysis of Human ACE2 expression. Kidney (K), lung (L) and brain **(B)** tissues were collected from the indicated transgenic hemizygous (HEM); homozygous (HOM) and wild type (WT) rats. **(A,D)** F344-Tg(CAG-*ACE2*)057Bryd; **(B,E)** SD-Tg(*ACE2*)955CPBryd; **(C,F)** SD-Tg(*ACE2*)058CVBryd line. **(A–C)** RT-PCR analysis. The rat reference gene *Hprt1* was used to normalize human *ACE2* mRNA expression; each bar represents expression for an individual rat. Error bars represent the standard deviations between triplicate technical replicates. **(D–F)** Western blot analysis., HepG2 cells (PC) were used as a positive control and Rat Hprt was used as the loading control. M is the molecular size standard lane with the sizes of the standards indicated in kDa.

RT-PCR was then used to detect expression of human ACE2 mRNA in the lung, brain, and kidney of F344-Tg(CAG-ACE2)057 rats (Figure 1A). The expression of human ACE2 mRNA was seen in all three tissues. Similar analysis detected mRNA expression in the lung and kidney of the SD-Tg(ACE2)955CPBryd line and in the kidney of the SD-Tg(ACE2)058CVBryd line but did not show ACE2 mRNA expression in the brain for either (Figures 1B, C). No human ACE2 was detected in tissues from wild type (WT) rats, including WT littermates, confirming the species specificity of the assay. Western blot analysis was performed to confirm protein expression (Figures 1D–F). Expression of the protein recapitulated the mRNA findings such that while human ACE2 protein expression was detectable in kidney and lungs from transgenic but not wild type rats, only F344- Tg(CAG-ACE2)057 transgene positive rats had detectable ACE2 protein in the brain. These findings are consistent with human ACE2 protein expression levels as reported in the Human Protein Atlas where ACE2 is expressed most highly in kidney, has low expression in respiratory system tissues (nasopharynx and bronchus) and no expression in brain (Human Protein Atlas - ACE2, n.d.; Uhlén et al., 2015).

### Susceptibility of *ACE2* rats to pulmonary challenge with SARS-CoV-2

To determine if rats expressing ACE2 are susceptible to SARS-CoV-2, groups of from male and female F344-Tg(CAG- *ACE2*)057Bryd, SD-Tg(*ACE2*)955CPBryd, SD-Tg(*ACE2*)058CVB ryd rats were challenged with intranasal instillation of 2.41 × 10^4^ PFU of the SARS-CoV-2 strain USA-WA1/2020 (Table 2). Genotypes for all rats were verified prior to challenge, and littermates were used as wild type (WT) controls. Animals were monitored for the development of clinical signs of disease for 10 days. All WT (transgene negative) animals from all three rat strains/stocks remained clinically normal for the duration of observation. Similarly, all transgene positive (hemizygous or homozygous) animals from the SD-Tg(*ACE2*)955CPBryd and SD-Tg(*ACE2*)058CVBryd remained clinically normal. In contrast, all hemizygous and homozygous F344-Tg(CAG-*ACE2*)057Bryd animals developed clinical symptoms that followed a similar progression to lethal disease. Following challenge, all rats were initially bright, alert and reactive for several days, followed by mild lethargy, slight fur ruffling, and the presence of increasing perinasal and/or periocular porphyrin accumulation indicative of lack of grooming. This developed into increased lethargy with evidence of hindlimb weakness or loss of coordination followed by hindlimb paralysis. A moribund state, as indicated by being fully prone with or without agonal breathing, generally developed within 12 h of hindlimb paralysis, between day 6 and day 7 post infection.

**TABLE 2:** Survival at high dose^1^ SARS-CoV-2 intranasal challenge.

Strain name	WT	HEMI	HOM
F344-Tg(CAG-*ACE2*)057Bryd	6/6	0/10	0/2
SD-Tg(*ACE2*)955CPBryd	4/4	28/28	3/3
SD-Tg(*ACE2*)058CVBryd	2/2	14/14	6/6

^1^2.41 × 10^4^ PFU per animal.

**FIGURE 2:**
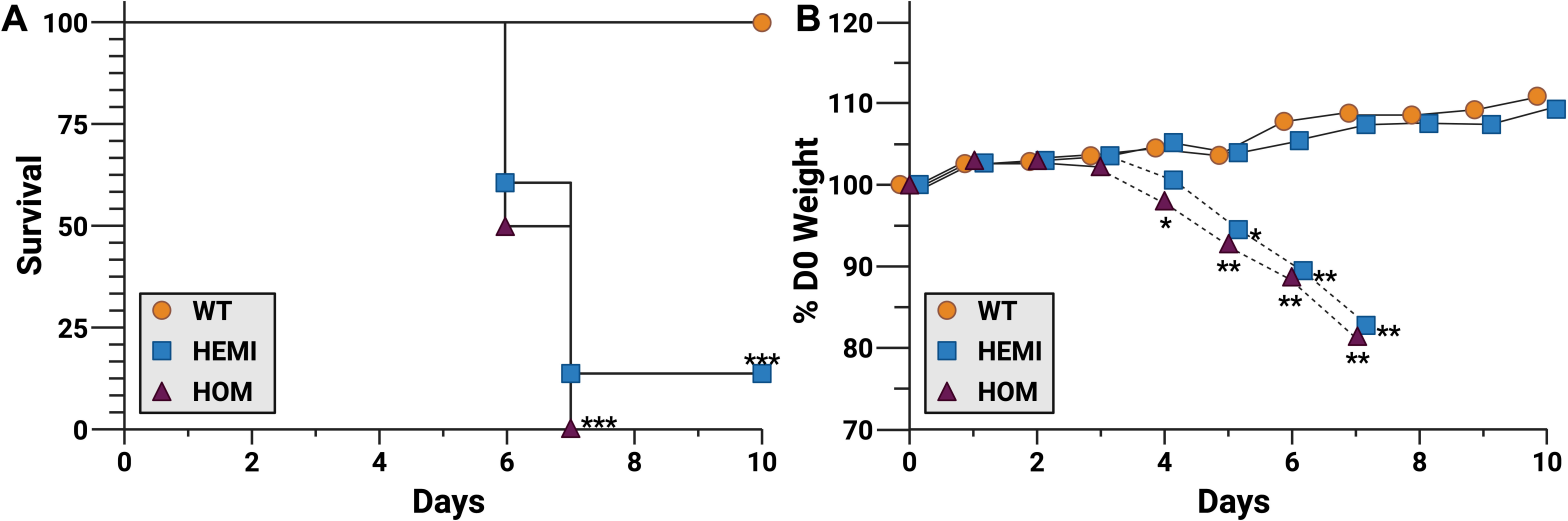
F344-Tg(CAG-*ACE2*)057Bryd transgenic rats are susceptible to SARS-CoV-2 challenge. Groups of F344-Tg(CAG-*ACE2*)057Bryd littermates without the *ACE2* transgene (wild type, WT, orange circles), or hemizygous (HEMI, blue squares) or homozygous (HOM, purple triangles) for transgene expression were challenge with SARS-CoV-2 by intranasal instillation of 14 PFU of the USA-WA1/2020 variant. Rats were monitored for survival **(A)** and clinical symptoms including weight loss **(B)** for up to 10 days. Weight loss of hemizygous animals has been separated into those that survived (solid lines, *n* = 2) and those that succumbed to disease (dashed lines, *n* = 10). Animals identified as moribund were humanely euthanized, and tissues were collected for histological analysis (see Figures 3, 4). *N* = 7 (WT), 13 (HEMI), and 6 (HOM) collected in two independent experiments. Survival data was analyzed by Mantel-Cox (log-rank) test. Daily change in weight was analyzed by ANOVA with Dunn’s multiple comparison test. *P < 0.05, **P < 0.01, ***P < 0.001 relative to WT. Survival of hemizygous and homozygous groups were not significantly different from each other.

To determine if there was a dose response to SARS-CoV-2 challenge in the F344-Tg(CAG-ACE2)057Bryd rats, groups of wild type, hemizygous, and homozygous male and female rats were challenged with 14 PFU of SARS-CoV-2 USA-WA1/2020 (Figure 2). As expected, transgene-negative animals (WT) were not susceptible to this lower challenge dose. Transgene-positive animals had significantly greater instances of clinical disease progressing to 84.6% (11/13 hemizygous rats) or 100% (6/6 homozygous) mortality (Figure 2A). Clinical signs developed in conjunction with progressing weight loss (Figure 2B) while WT littermates gained weight over the challenge period. Individual animal weights from the day of infection to endpoint (moribund or end of observation period) are in Supplementary Table 1. Weight loss and clinical symptoms were perfectly correlated, and hemizygous transgenic rats that did not develop other clinical signs of disease gained a similar amount of weight to WT animals. Interestingly, both hemizygous animals that did not show signs of disease were female.

### Tissue damage due to intranasal infection with SARS-CoV-2 in F344-Tg(CAG-*ACE2*)057Bryd rats

**FIGURE 3:**
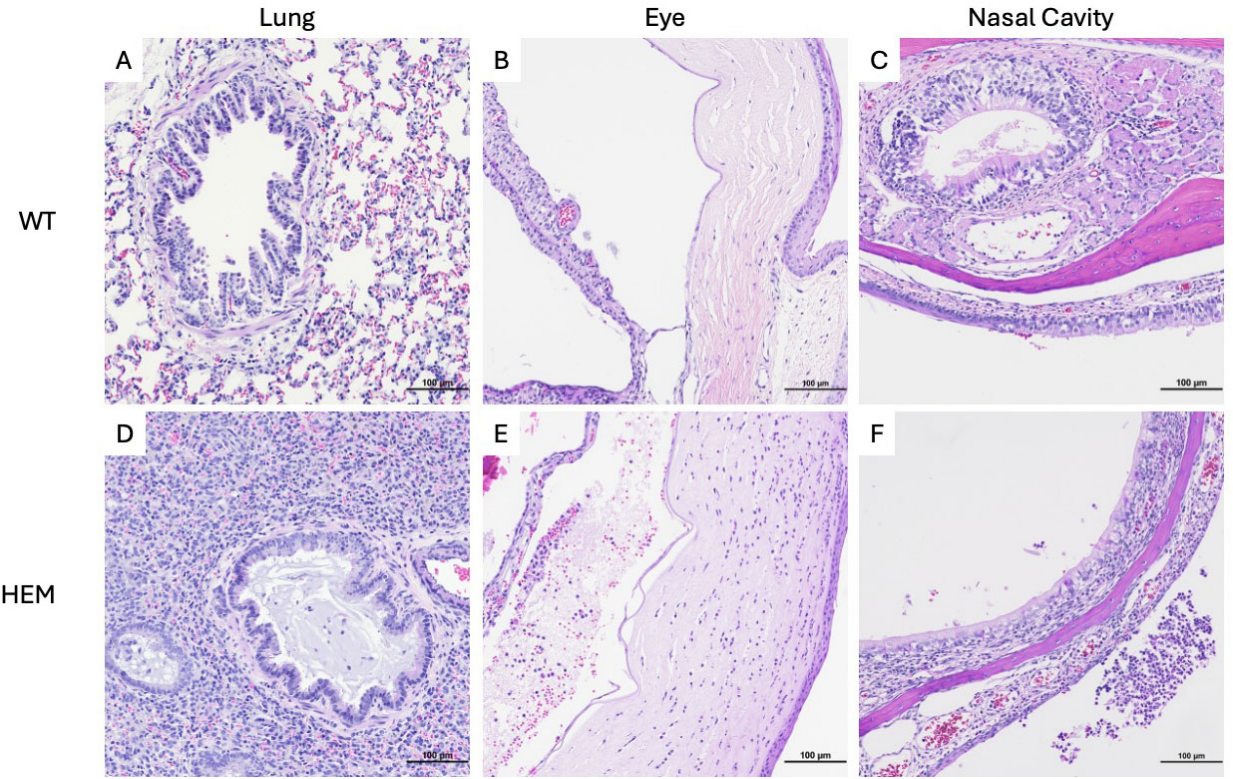
Tissue damage of F344-Tg(CAG-*ACE2*)057Bryd rats challenged with SARS-CoV-2. Sections of lung **(A,D)**, eye **(B,E)** and nasal cavity **(C,F)** from wild type **(A–C)** and F344-Tg(CAG-*ACE*2)057Bryd hemizygous rats infected with either high **(D)** or low **(E,F)** doses of SARS-CoV-2 stained with hematoxylin and eosin. **(D)** One hemizygous rat infected with the high dose demonstrates broncho and interstitial pneumonia. Bronchiolar lumina are multifocally filled with mucinous material and low numbers of neutrophils; alveolar septae and lumina contain alveolar macrophages, lymphocytes and mild hemorrhage. **(E)** Hemizygous rats infected with the low dose show anterior uveitis characterized by infiltration of the iris and choroid body with neutrophils and lymphocytes and anterior chamber accumulations of eosinophilic flocculent material, neutrophils, few lymphocytes and red blood cells. The corneal stroma is infiltrated by moderate numbers of neutrophils. **(F)** Hemizygous rats infected with the low dose show foci of epithelial erosion and associated mucosal neutrophilic infiltrates as well as accumulation of suppurative exudate in the nasal cavity. All images taken at 200× magnification.

To better understand the pathology of SARS-CoV-2 in susceptible transgenic rats, tissues were collected for histology on day 6 post-inoculation and from moribund animals. To assess putative primary sites of infection, the upper and lower respiratory tract, sections of nasal cavity (low dose group only) and lung (both challenge doses) were stained with H&E (Figure 3). All challenged rats, including WT, had mild hyperplasia of peribronchiolar lymphoid tissue and mild multifocal perivascular and/or subpleural lymphoid aggregates consistent with common background lesions of rats. Inoculation-associated lung lesions in most transgenic rats were characterized by very mild focal to multifocal interstitial pneumonia with alveolar macrophage infiltrates and interstitial lymphoid infiltrates. One rat infected with the high dose also exhibited moderate bronchointerstitial pneumonia with alveolar microhemorrhages, type II pneumocyte hyperplasia, and bronchiolar mucoid exudate containing few neutrophils (Figure 3D). No lesions of this type were seen in the alveolar parenchyma or bronchioles of WT rats (Figure 3A). The nasal cavities of transgene positive rats inoculated with the low dose had mild to moderate neutrophilic and necrotizing rhinitis in the most rostral portion of the cavity. These lesions included epithelial necrosis with foci of hemorrhage and associated mucosal neutrophilic infiltrates and accumulation of suppurative exudate in the nasal cavity (Figure 3F). In nasal cavities of low dose-infected WT rats, occasional lymphocytes were seen migrating through mucosal epithelium of the vomeronasal organ and nasal cavity, but no necrotizing lesions or luminal exudate were seen (Figure 3C).

Eyes of low dose-infected rats were also examined, and all hemizygous rats showed mild to moderate neutrophilic and lymphocytic anterior uveitis, with two rats having moderate keratitis (Figure 3E). Very mild iritis was seen in one WT rat (data not shown).

**FIGURE 4:**
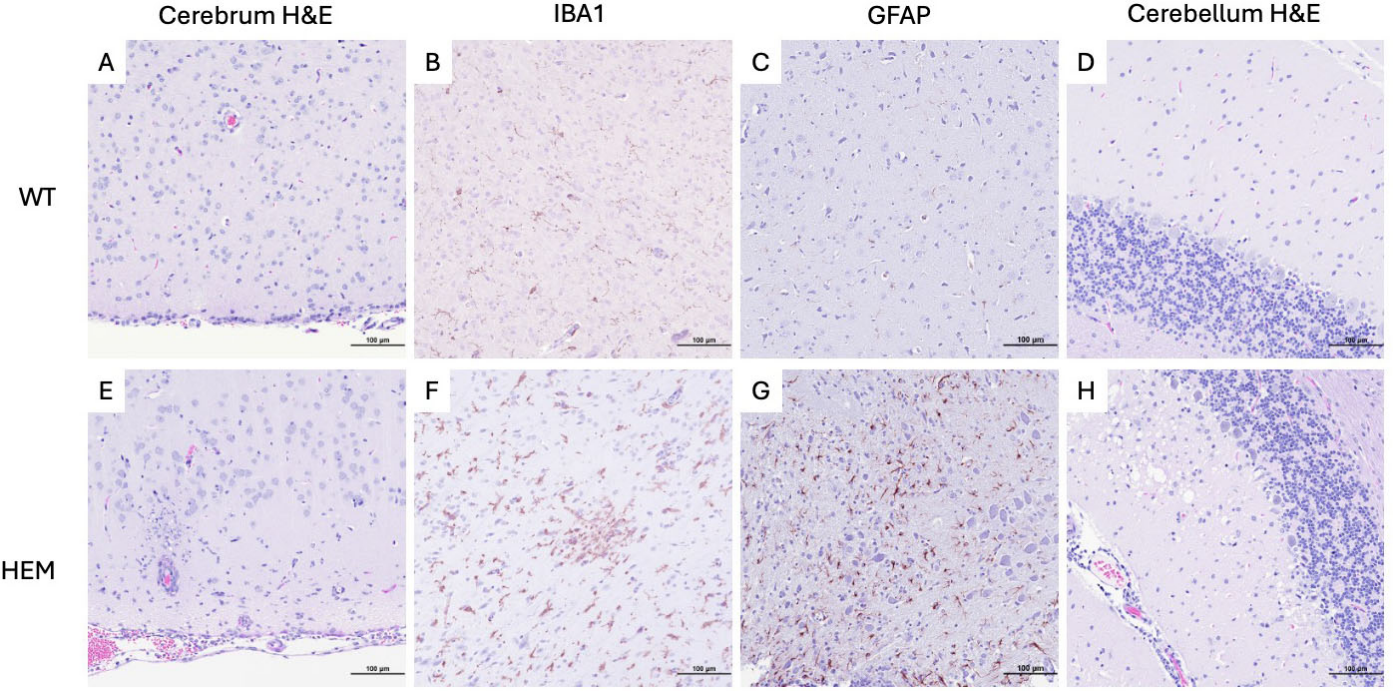
Cellular changes in the brains of F344-Tg(CAG-*ACE2*)057Bryd rats that succumbed to a high dose SARS-CoV-2 challenge. Sections of cerebrum and cerebellum from wild type **(A–D)** and F344-Tg(CAG-*ACE2*)057Bryd hemizygous rats **(E–H)** infected with SARS-CoV-2 stained with hematoxylin and eosin **(A,D,E,H)** or subjected to IBA **(B,F)** or GFAP **(C,G)** immunohistochemistry. **(E)** Histology of hemizygous rats exhibit macrophagic meningitis, perivascular cuffing, encephalitis and necrosis. **(F)** IBA IHC demonstrates marked glial cell activation. **(G)** GFAP IHC shows astrocyte proliferation swelling in areas of gliosis. **(H)** Cerebellum exhibits Purkinje cell loss associated with vacuolated neuropil of the adjacent molecular layer and scattered pyknotic and karyorrhectic debris (apoptosis as determined by PCNA IHC) with mildly increased numbers of astrocytes or microglia. All images taken at 200× magnification.

It has been established that SARS-CoV-2 infection is associated with neuropathology in the widely used B6.Cg-Tg(K18-*ACE2*)2Prlmn/J transgenic mouse due to the ubiquity of the K18 promoter in this model (Oladunni et al., 2020; Carossino et al., 2022; Vidal et al., 2022). To understand if morbidity and mortality similarly correlated with neurologic disease in the F344-Tg(CAG-*ACE2*)057Bryd transgene positive rats, H&E-stained sections of brain were examined (Figure 4). No lesions were seen in the brains of WT rats. However, in transgenic rats given a low dose of virus, mild meningeal lymphocytic infiltrates and perivascular lymphoid cuffs were noted. The latter were associated with mild parenchymal gliosis in some locations. Rats inoculated with a high dose of virus showed more severe brain lesions (Figure 4E). In these animals, cerebral lesions were most evident in the hindbrain and brainstem where moderate multifocal to diffuse encephalitis characterized by ghosting (loss of nuclear detail and Nissl substance) of neuronal cell nuclei, gliosis and scattered apoptotic debris was seen. To better understand the cellular involvement in the brain, these tissues were analyzed by immunohistochemistry (IHC) for calbindin (Purkinje cells), CD3 (lymphocytes), PAX5 (B cells), IBA1 (microglial cell activation), GFAP (astrocytes), and PCNA (apoptosis). Marked microglial cell proliferation was confirmed by IBA1 IHC (Figure 4F). Astrocyte proliferation and activation were also evident in these areas with GFAP IHC that demonstrated swelling of astrocyte nuclei with margination of chromatin and exacerbation of typical branching pattern (Figure 4G). Small vessels within areas of encephalitis were often cuffed by mononuclear cells with fewer numbers of neutrophils (Figure 4E). Mononuclear cells were presumably monocytes or macrophages based on negative CD3 and PAX5 IHC (data not shown). Meningeal infiltrates included lymphocytes, CD3-negative mononuclear cells and fewer numbers of neutrophils (data not shown).

In the cerebellum of F344-Tg(CAG-*ACE2*)057Bryd hemizygous rats, there was a moderate segmental loss of Purkinje cells confirmed by calbindin IHC. These areas of Purkinje cell loss were often associated with vacuolated neuropil of the adjacent molecular layer and scattered pyknotic and karyorrhectic debris (apoptosis as determined by PCNA IHC) with mildly increased numbers of astrocytes or microglia (Figure 4H). The white pulp of the cerebellar stalk also showed mild multifocal vacuolation indicative of degeneration.

## Discussion

Our goal was to generate rats that expressed human *ACE2*. The rationale for the choice of promoter in our transgene design was to provide flexibility in *ACE2* expression levels: potential over-expression (CAG promoter) or physiological expression (human *ACE2* promoter). We chose a random transgenesis approach for ease of genetic engineering. We chose not to knock-in our constructs into the endogenous rat *ACE2* locus to avoid perturbation of native rat *ACE2* expression or function. Expression of ACE2 was consistent with the promoter used: the strain with the CAG promoter had more ubiquitous expression, including expression in brain where ACE2 is not normally expressed whereas the lines with the human *ACE2* promoter appeared to more faithfully recapitulate expected human ACE2 expression patterns such that expression was highest in kidney, low in lung and not seen in brain. For the two lines with the *ACE2* promoter driving expression, the level of expression appeared to correspond to the number of transgene copies with SD-Tg(*ACE2*)955CPBryd (30 copies) having higher ACE2 expression than SD-Tg(*ACE2*)058CVBryd (8) in the tissues examined. F344-Tg(CAG-*ACE2*)057Bryd had similar expression levels in the tissues examined as expected given the ubiquitously expressed CAG promoter.

We show here that a single copy of human *ACE2* driven by the ubiquitously expressed CAG promotor is sufficient to drive lethal susceptibility to infection with SARS-CoV-2 in F344 transgenic rats. This is in marked contrast with transgene-negative littermates. Our results suggest that SARS-CoV-2 infection in F344-Tg(CAG-*ACE2*)057Bryd rats is associated with an upper respiratory tract infection that does not progress to severe pneumonia. Moreover, the findings of ocular lesions suggest that virus colonizing the nasal cavity can spread via the nasolacrimal duct with transgene positive rats being susceptible to associated lesion development. Additional studies to demonstrate the presence of replicative virus would need to be performed to confirm these findings.

Rather than pneumonia, rats infected with a high dose of virus likely succumbed to neurological complications as evidenced by moderate meningoencephalitis seen in these rats. These histological findings are consistent with the exhibited clinical signs. Neuroinvasion of SARS-CoV-2 in the B6.Cg-Tg(K18-*ACE2*)2Prlmn/J transgenic mouse and subsequent encephalitis is thought to begin with initial infection of neurons in the sinus cavity that allows spread of the virus into the olfactory bulb and from there to additional regions of the CNS (Oladunni et al., 2020; Carossino et al., 2022). However, breakdown of the blood-retinal barrier has also been observed, and blood-brain barrier breach is also a possible mechanism (Monu et al., 2024). Collectively, our data associate brain lesions with death in transgenic rats. Though breaching the blood-brain barrier cannot be ruled out, the finding of rhinitis suggests a similar mechanism of neuroinvasion as observed in the B6.Cg-Tg(K18-*ACE2*)2Prlmn/J transgenic mouse.

Collectively, these findings suggest that these rats will be useful for studies of the mechanisms of neurological complications of COVID, including preclinical therapeutic-efficacy studies, as well as molecular host CNS-virus interaction studies. Conversely, neurologic disease may be unwanted in some studies, and this model could be further refined by using aerosol challenge which may result in more diffuse/less bolus deposition of virus.

Despite evidence of *ACE2* expression, transgene positive SD-Tg(*ACE2*)955CPBryd and SD-Tg(*ACE2*)058CVBryd rats showed a surprising lack of susceptibility to SARS-CoV-2 challenge. One major difference between these lines and the susceptible F344-Tg(CAG-*ACE2*)057Bryd was the lack of *ACE2* expression in the brain in the former lines. This is likely due to promotor-driven expression differences. Another possible explanation for lack of susceptibility could be a genetic difference related to the different strain/stock backgrounds (F344 versus SD) of our rat lines. Perhaps the outbred SD genetic background confers some genetic resistance to infection. Wild type SD rats have been shown to be competent for viral replication, so it is unlikely that viral replication is arrested in these animals, although it should be acknowledged that the ancestral isolate used in our study was not examined in the study by Wang et al. (2023). It remains possible that the presence of modifiers in the SD genetic background play a role in the lack of clinical signs in these rat models. Although beyond the scope of the current study to verify, we propose that the SD-Tg(*ACE2*)955CPBryd and SD-Tg(*ACE2*)058CVBryd lines might be consistent with typical infection in healthy adult humans, and that this model may be appropriate for understanding the impact of comorbidities or as a model of post-acute sequelae of COVID (PASC).

We anticipate that all three of these lines may be useful for studying various aspects of other coronaviruses that utilize the ACE2 receptor for cellular entry, including SARS-CoV (Gallagher and Buchmeier, 2001). While MERS virus uses dipeptidyl peptidase-4 (DPP4) as an entry receptor, other coronaviruses also found in bats, including NeoCoV, PDF-2180, and the human alpha coronavirus HCoV NL63 can use ACE2 through RBD-Spike interactions (Li et al., 2007; Xiong et al., 2022). This suggests that humanized ACE2 models may continue to be useful for zoonotic infections with the potential to crossover into human disease.

## Data Availability

The original contributions presented in this study are included in this article/Supplementary material, further inquiries can be directed to the corresponding author.
