# Supplementary material for: Generation and characterization of a humanized ACE2 rat model for the study of SARS-CoV-2 and COVID-19
Source: Front Microbiol. 2025 Oct 29;16:1680273. doi: 10.3389/fmicb.2025.1680273 (PMC12605091; doi:10.3389/fmicb.2025.1680273)
Supplement: Supplementary file 1 [file Table_1.docx]

**Supplemental Table S1. Individual Animal Endpoint and Weight from Figure 3**

| ***Animal ID*** | ***Genotype*** | ***Sex*** | ***Day 0 weight*** | ***Endpoint^3^*** | ***Endpoint Weight*** |
| --- | --- | --- | --- | --- | --- |
| 122CP | WT | F | 156 | 10 | 168 |
| 123CP | WT | F | 151 | 10 | 161 |
| 124CP | WT | F | 156 | 10 | 168 |
| 055CP | WT | M | 205 | 10 | 235 |
| 127CP | WT | M | 203 | 10 | 228 |
| 119CP | WT | M | 212 | 10 | 240 |
| 120CP | WT | M | 207 | 10 | 239 |
| 131CP | HEMI | F | 130 | 6 | 121 |
| 134CP | HEMI | F | 143 | 6 | 133 |
| 056CP | HEMI | F | 145 | 7 | 119 |
| 058CP | HEMI | F | 136 | 7 | 111 |
| 132CP | HEMI | F | 131 | 7 | 114 |
| 133CP | HEMI | F | 135 | 7 | 110 |
| 121CP | HEMI | F | 148 | 10 | 162 |
| 125CP | HEMI | F | 144 | 10 | 158 |
| 126CP | HEMI | M | 202 | 6 | 174 |
| 130CP | HEMI | M | 204 | 6 | 182 |
| 117CP | HEMI | M | 204 | 6 | 177 |
| 128CP | HEMI | M | 203 | 7 | 174 |
| 129CP | HEMI | M | 203 | 7 | 185 |
| 062CP | HOM | F | 132 | 6 | 117 |
| 135CP | HOM | F | 132 | 6 | 117 |
| 057CP | HOM | F | 150 | 7 | 124 |
| 061CP | HOM | F | 142 | 7 | 115 |
| 054CP | HOM | M | 229 | 6 | 200 |
| 118CP | HOM | M | 217 | 7 | 177 |

^3^Animals were euthanized at humane endpoint (D6-D7 post challenge) or at the experimental endpoint (D10), no animals that reached experimental endpoint showed any signs or symptoms of the challenge.
